# An Equity‐Focused Systematic Analysis of Antimicrobial Resistance National Action Plans in 14 West African Countries

**DOI:** 10.1111/tmi.70037

**Published:** 2025-09-20

**Authors:** Yusuff Adebayo Adebisi, Isaac Olushola Ogunkola, Adeola Bamisaiye, Aminat Olaitan Adebayo, Noah Sesay, Kwasi Yelarge, Don Eliseo Lucero‐Prisno

**Affiliations:** ^1^ Global Health Focus Abuja Nigeria; ^2^ College of Social Sciences, University of Glasgow Glasgow UK; ^3^ Nuffield Department of Population Health University of Oxford Oxford UK; ^4^ Centre for Tropical Medicine and Global Health University of Oxford Oxford UK; ^5^ Department of Agricultural Extension and Rural Development, Faculty of Agriculture University of Ibadan Ibadan Nigeria; ^6^ Ministry of Health of Sierra Leone Ola During Children's Hospital Freetown Sierra Leone; ^7^ Faculty of Pharmacy and Pharmaceutical Sciences Kwame Nkrumah University of Science and Technology Kumasi Ghana; ^8^ Department of Global Health and Development London School of Hygiene and Tropical Medicine London UK

**Keywords:** antimicrobial resistance, ECOWAS, health equity, health governance, National Action Plans, vulnerable populations, West Africa

## Abstract

**Objectives:**

Antimicrobial resistance is a growing global health threat that disproportionately affects socially and economically disadvantaged populations. National Action Plans are critical for coordinating national responses, but the extent to which they address equity remains unclear. This study assessed how antimicrobial resistance National Action Plans from 14 West African countries incorporate equity considerations.

**Methods:**

We reviewed antimicrobial resistance National Action Plans from 14 West African countries using a four‐domain equity framework: (1) recognition of equity, (2) identification of vulnerable populations, (3) inclusion of equity‐oriented interventions and (4) integration of equity into governance and monitoring. We assessed whether National Action Plans acknowledged 16 high‐risk groups, including people living with HIV, displaced or mobile populations, children and adolescents, older adults, people with mental health disorders, rural residents, people with chronic illnesses, people living with disabilities, pregnant women, low‐income populations, healthcare workers, people with substance use disorders, incarcerated populations, indigenous or minority groups, homeless populations and migrants or seasonal workers.

**Results:**

All National Action Plans adopted a One Health approach, but equity was inconsistently addressed. Most did not explicitly reference equity, and none included equity‐related indicators in monitoring frameworks. Healthcare workers and rural populations were the most frequently mentioned groups. Common interventions included hygiene promotion, public awareness campaigns and training of healthcare workers, but these were largely generic and rarely adapted to the specific needs of marginalised populations. Stakeholder engagement was often multisectoral but seldom ensured the participation of disadvantaged groups. Across the region, the lack of disaggregated data and tailored strategies highlights a significant equity gap.

**Conclusion:**

Equity remains insufficiently integrated into antimicrobial resistance governance in West Africa. Future National Action Plans must explicitly identify at‐risk populations, include equity indicators and involve affected communities in planning and oversight. Embedding equity is essential to building resilient and people‐centred antimicrobial resistance strategies.

## 1 Introduction

Antimicrobial resistance (AMR) is one of the most pressing global health challenges of the 21st century. It threatens the effectiveness of essential medical treatments and risks reversing decades of progress in infectious disease control [1, 2, 3]. In 2019, bacterial AMR was directly responsible for an estimated 1.27 million deaths worldwide and contributed to approximately 4.95 million deaths overall, with the burden falling disproportionately on low‐ and middle‐income countries (LMICs), particularly in sub‐Saharan Africa [4]. Within the World Health Organization (WHO) African Region, AMR was associated with an estimated 1.05 million deaths (95% uncertainty interval [UI]: 829,000–1,316,000) and directly caused 250,000 deaths (UI: 192,000–325,000) in the same year [5]. In West Africa, the challenge of AMR containment is intensified by weak health systems, a high burden of infectious diseases and the widespread use of antibiotics in unregulated and informal markets [6, 7]. The rising prevalence of resistant pathogens in the region reflects microbial adaptation as well as entrenched structural inequalities related to healthcare access, sanitation, governance and pharmaceutical regulation [8, 9]. Addressing AMR in this context therefore requires technical interventions as well as equity‐focused strategies that consider the social and systemic drivers of resistance.

To guide national responses, WHO launched the Global Action Plan (GAP) on AMR in 2015, urging all member states to develop National Action Plans (NAPs) aligned with its five strategic objectives [10]. These include improving awareness, strengthening surveillance, reducing infection, optimising antimicrobial use and ensuring sustainable investment. Since then, countries within the Economic Community of West African States (ECOWAS) have made progress in formulating and publishing AMR NAPs. However, little is known about whether and how these NAPs incorporate equity, despite increasing calls to integrate equity considerations into health policy design and evaluation.

Recent analyses have examined NAPs more broadly. For example, Charani et al. reviewed more than 100 NAPs and identified important implementation and optimisation gaps across policy, surveillance, prescribing systems and public engagement [11]. While this study provided critical breadth, it did not specifically interrogate equity or regional disparities in depth. Our work builds on this evidence by offering the first systematic, equity‐focused analysis of AMR NAPs in West Africa. By applying a structured analytic framework, we assess the extent to which these NAPs recognise vulnerable and marginalised populations, propose tailored interventions and embed equity in governance and monitoring systems.

AMR disproportionately affects groups such as people living with HIV, displaced communities, rural residents, the elderly and children [12, 13]. These populations often face structural barriers to timely diagnosis, appropriate antimicrobial treatment and infection prevention services. In the context of the Sustainable Development Goals (SDGs), particularly the pledge to “leave no one behind,” assessing the equity responsiveness of AMR NAPs is essential for effective implementation. This analysis, therefore, aims to generate actionable insights to inform future iterations of AMR strategies in West Africa and other LMICs.

## 2 Methods

### 2.1 Study Design

This study employed a qualitative document review methodology to conduct a systematic, equity‐focused analysis of NAPs on AMR across 14 countries in the ECOWAS. This approach aligns with recent guidance advocating for the application of equity‐oriented frameworks in health policy research and global health governance [14, 15].

### 2.2 Selection of Countries and Documents

**FIGURE 1:**
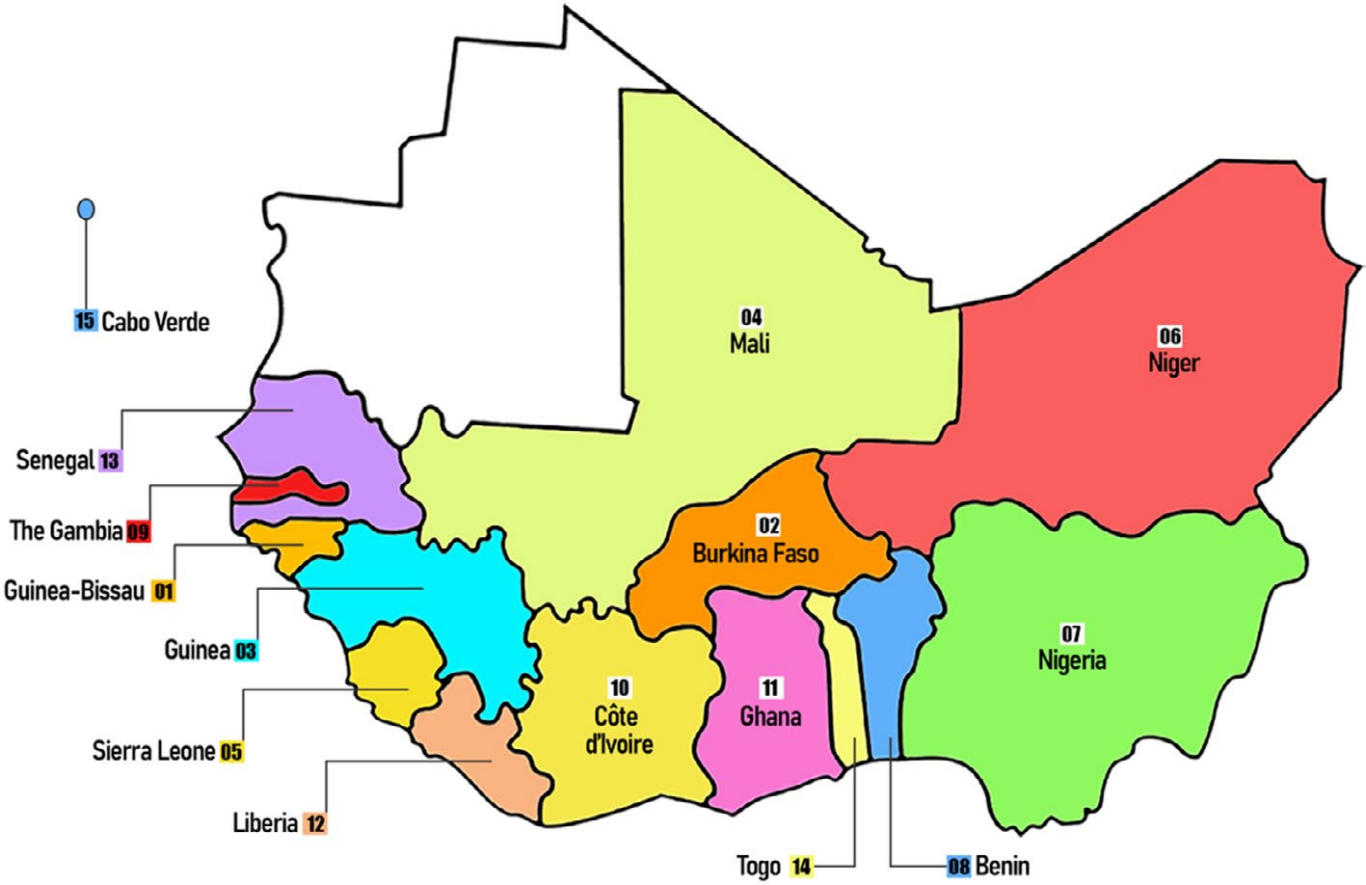
Geographic map of West Africa highlighting the 14 ECOWAS countries. Fourteen countries with publicly accessible AMR National Action Plans (NAPs) were included in this review. The Gambia was excluded due to the absence of a publicly accessible NAP at the time of analysis.

This study included 14 member states of the ECOWAS that had publicly available NAPs on AMR as of August 2025. ECOWAS comprises 15 countries; however, The Gambia was excluded from this review due to the absence of publicly accessible AMR NAPs. The 14 countries included in the analysis were: Benin, Burkina Faso, Cabo Verde, Côte d'Ivoire, Ghana, Guinea, Guinea‐Bissau, Liberia, Mali, Nigeria, Niger, Senegal, Sierra Leone and Togo (see Figure 1). Although Burkina Faso, Niger and Mali formally withdrew from ECOWAS on 29 January 2025 [16], their NAPs were included in this study because the three countries were active ECOWAS members at the time their plans were developed and published and their documents were publicly available. The inclusion of these plans ensures methodological consistency and regional comparability across ECOWAS states. NAPs were identified through a structured document search involving official Ministry of Health websites, the WHO AMR document repository [17], the Africa Centres for Disease Control and Prevention (Africa CDC) platform and direct communication with national AMR focal points. Only publicly available documents were included. Where multiple versions of a country's NAP were available, all versions were reviewed to capture changes over time in equity framing, strategic focus and implementation planning.

### 2.3 Analytic Framework

To guide the analysis of equity considerations in national AMR action plans, a structured data extraction framework was developed based on conceptual literature on inclusive health and social determinants of health [18], established models for equity in health policy analysis [14, 15] and a hypothesised list of vulnerable populations (see Table 1). The framework was designed to capture whether and how NAPs acknowledge, prioritise, or address the needs of marginalised groups who are disproportionately affected by AMR due to factors such as limited healthcare access, heightened exposure to infectious risks, or systemic social exclusion.

**TABLE 1:** Vulnerable and marginalised populations included in the equity framework.

Population group	Rationale for inclusion
People living with HIV	Immunocompromised; high infection risk; often stigmatised or excluded from mainstream care
Displaced or mobile populations (e.g., refugees, internally displaced persons, migrants)	Limited healthcare access; disrupted treatment; exposure to poor sanitation
Children and adolescents	Higher risk of infections; vulnerable immune systems; often overlooked in health planning
The elderly	Age‐related immunodeficiency; co‐morbidities; reduced mobility and access to services
People with mental health disorders	Often excluded from formal care; elevated infection risk due to institutionalisation or homelessness; may struggle with treatment adherence; face significant stigma
Rural residents	Limited access to healthcare facilities, diagnostics and safe antimicrobials
People with chronic illnesses	Increased infection risk; frequent healthcare exposure; often excluded from surveillance
People living with disabilities	Experience systemic exclusion; physical, sensory and cognitive barriers to healthcare and AMR messaging
Pregnant women	Immunological vulnerability; high antenatal care needs; maternal–neonatal transmission risk
Low‐income populations	Limited affordability of antimicrobials; poor housing, sanitation and nutrition
Healthcare workers	High exposure to antimicrobial‐resistant pathogens due to their frontline role; require inclusion in training, infection prevention measures and occupational protections
People with substance use disorders	Often excluded from formal care; elevated risk of infections and treatment non‐compliance
Incarcerated populations (e.g., people in prisons or jails)	Live in overcrowded environments with poor sanitation and limited access to healthcare; high transmission risk of resistant infections (e.g., TB)
Indigenous or minority populations	Face systemic exclusion; underrepresented in surveillance and public health programming
Homeless populations and slum dwellers	Vulnerable to infections; limited hygiene and healthcare access
Migrants and seasonal workers	High mobility; poor continuity of care; language and documentation barriers

While other widely used frameworks, such as Walt and Gilson's Policy Triangle, provide valuable tools for analysing policy processes, actors and political context, our focus was on the content of NAPs rather than on policy development dynamics. For this reason, we adopted an equity‐focused framework that enabled a systematic appraisal of whether NAPs explicitly recognise equity, identify vulnerable populations, propose tailored interventions and incorporate equity into governance and monitoring. This approach was therefore more closely aligned with the study objectives, which centred on understanding how equity is embedded within AMR policy design in West Africa.

The final analytic framework comprised four interrelated domains. The first domain, *recognition of equity*, assessed whether the NAP explicitly referred to concepts such as equity, inclusion, vulnerable or marginalised populations, or social determinants of health. The second domain focused on the *identification of vulnerable and marginalised groups*, evaluating whether the plan acknowledged the needs of priority populations. These groups were included on the basis of either epidemiological vulnerability or structural barriers to prevention and care. The third domain, *equity‐responsive interventions*, examined whether the NAP proposed targeted programmes or strategies specifically designed to reduce disparities in AMR‐related risks or outcomes. This included actions such as community‐based awareness campaigns, rural health outreach, or tailored infection prevention strategies for high‐risk populations. The fourth and final domain assessed *equity in governance and monitoring*, looking for mechanisms that promote inclusive stakeholder engagement, such as the participation of civil society or patient advocacy groups and the presence of equity‐relevant indicators within monitoring and evaluation frameworks.

### 2.4 Analysis

Each NAP was reviewed in full by four independent coders using a standardised extraction template to ensure consistent assessment across the four equity domains. For documents not written in English, primarily those in French or Portuguese, coders fluent in the relevant language conducted the primary review. To enhance accuracy and minimise misinterpretation, key passages in these non‐English documents were also cross‐checked using Google Translate. Any coding discrepancies were resolved through consensus discussions, with input from additional reviewers or native speakers when necessary. Following document review, we generated summary tables to present country‐level variation across the equity domains. A narrative synthesis approach was used to interpret patterns and draw comparisons across countries. To further strengthen analytical rigour, an additional independent review was conducted by two researchers with expertise in health policy and social determinants of health. In addition, the research team developed a set of recommendations to support more inclusive NAPs, which were further reviewed by five independent experts in AMR for their feedback and suggestions on the need to better address the needs of marginalised and vulnerable populations. A reflexive approach was adopted throughout, including regular team debriefings to reflect on researcher positionality and the political or institutional contexts influencing how equity was addressed in each NAP.

## 3 Results

### 3.1 AMR Burden and the Case for Equity in West Africa

The 2019 burden of AMR across 14 West African countries shows substantial cross‐country variation in mortality rates and pathogen‐specific drivers (Table S1) [5]. Nigeria, Mali, Burkina Faso, Niger and Guinea recorded the highest numbers of AMR‐attributable deaths in the region, in several cases exceeding mortality from other major health threats such as malaria, tuberculosis, and maternal and neonatal disorders [5]. In contrast, countries such as Cabo Verde and Liberia reported comparatively lower burdens, though AMR‐related mortality in these settings still surpassed several other leading causes of death. Across the region, the leading pathogens contributing to AMR mortality included *Klebsiella pneumoniae, Streptococcus pneumoniae, Escherichia coli, Staphylococcus aureus* and *Pseudomonas aeruginosa* , with respiratory, bloodstream, intraabdominal and central nervous system infections being the most common clinical manifestations. Despite differences in overall magnitude, common themes emerged, including rural exclusion, health system fragility, conflict and displacement and persistent socioeconomic and geographic disparities. These findings indicate that AMR disproportionately affects marginalised populations and underline the need for equity‐informed responses that explicitly address the structural determinants driving unequal outcomes across West Africa.

### 3.2 Overview of AMR NAPs Across 14 ECOWAS Countries

Across the 14 West African countries reviewed, all had developed NAPs to address AMR, with publication years ranging from 2017 to 2024. These plans vary in structure and emphasis but uniformly reflect the growing adoption of the One Health approach, integrating human, animal and environmental sectors. Ministries of Health typically serve as lead agencies, often in collaboration with agricultural and environmental authorities. While the language of publication aligns with national official languages (English, French, or Portuguese), all NAPs include key strategic pillars such as AMR surveillance, laboratory strengthening, public awareness and antimicrobial stewardship. Notably, Nigeria has launched a second‐generation plan (NAP 2.0) to build on the progress of its initial 2017–2022 framework. Table 2 summarises the main characteristics of each country's NAP, including responsible institutions and strategic focus areas.

**TABLE 2:** Characteristics of the antimicrobial resistance National Action Plan in the 14 West African countries.

Country	Title of the action plan	Language of publication	Responsible ministry/agency	Specific focus	National action plans reference
Burkina Faso	Plan d'Action National Multisectoriel de Lutte Contre la Résistance aux Antimicrobiens 2017–2020	French	Ministry of Health, Ministry of Animal Resources, Ministry of Agriculture and Ministry of Environment	Strengthening AMR surveillance and laboratory systems, promoting rational use of antimicrobials in human and animal health, raising public awareness and enhancing multisectoral coordination and policy enforcement	https://www.who.int/publications/m/item/burkina‐faso‐national‐multisectoral‐strategic‐plan‐to‐combat‐antimicrobial‐resistance‐(french)
Cabo Verde	Plano Nacional de Luta Contra a Resistência Antimicrobiana 2018–2022	Portuguese	Ministry of Health and Social Security (MSSS), Ministry of Agriculture and Environment (MAA)	Surveillance of AMR, strengthening laboratory capacity, public awareness, infection prevention and promoting rational use of antimicrobials in human, animal and environmental sectors through a One Health approach	https://www.who.int/publications/m/item/cabo‐verde‐nap‐2018‐2022
Côte d'Ivoire	Plan d'Action National pour la Résistance aux Antimicrobiens (PAN‐RAM Côte d'Ivoire) 2021–2025	French	Ministry of Health and Public Hygiene, Ministry of Agriculture and Rural Development, Ministry of Animal and Fisheries Resources, Ministry of Environment and Sustainable Development and Ministry of Higher Education and Scientific Research	Promote rational use of antimicrobials in human, animal and environmental health while strengthening surveillance and capacity‐building	https://www.who.int/publications/m/item/C%C3%B4te‐d'Ivoire‐national‐multisectoral‐action‐plan‐against‐antimicrobial‐resistance‐2021‐2025
Ghana	National Action Plan for Antimicrobial Use and Resistance 2017–2021	English	Ministry of Health, Ministry of Food and Agriculture, Ministry of Environment, Science, Technology and Innovation, Ministry of Fisheries and Aquaculture Development	Surveillance of AMR, strengthening laboratory capacities, public awareness and promoting “One Health” approach	https://www.who.int/publications/m/item/ghana‐national‐action‐plan‐for‐antimicrobial‐use‐and‐resistance
Guinea	Plan d'Action National Multisectoriel de lutte contre la Résistance aux Antimicrobiens 2020–2024	French	Ministère de la Santé (Lead), Ministère de l'Agriculture, Ministère de l'Environnement, Ministère de l'Élevage et des Productions Animales	Multisectoral approach to combat antimicrobial resistance (RAM) through One Health strategies, targeting human health, animal health, agriculture and the environment	https://www.who.int/publications/m/item/multisectoral‐national‐action‐plan‐against‐antimicrobial‐resistance‐2020‐2024
Niger	Plan d'action national de lutte contre la résistance aux antimicrobiens 2025–2027	French	Ministère de la Santé Publique; Ministère de l'Élevage; Ministère de l'Agriculture; Ministère de l'Environnement (via le Comité multisectoriel « Une seule Santé »)	A One Health strategy to strengthen AMR surveillance, promote rational antimicrobial use, enhance multisectoral governance, raise awareness and reinforce regulatory frameworks	https://www.who.int/publications/m/item/niger‐‐national‐action‐plan‐against‐antimicrobial‐resistance‐2025‐2027‐(french)
Guinea‐Bissau	Plano de Ação Nacional Contra a Resistência Antimicrobiana 2023–2028	Portuguese	Ministério da Saúde Pública (MINSAP) (Lead), Ministério da Agricultura, Ministério das Pescas, Ministério do Ambiente	Multisectoral “One Health” approach to combat antimicrobial resistance in human health, animal health, food production and the environment	https://www.who.int/publications/m/item/guinea‐bissau‐‐national‐action‐plan‐against‐antimicrobial‐resistance‐2023‐2028‐(portuguese)
Liberia	National Action Plan on Prevention and Containment of Antimicrobial Resistance 2018–2022	English	Ministry of Health (MoH), National Public Health Institute of Liberia (NPHIL)	Human, animal and environmental health under a One Health approach	https://www.who.int/publications/m/item/liberia‐national‐action‐plan‐on‐prevention‐and‐containment‐of‐antimicrobial‐resistance
Mali	Plan d'Action National (PAN) de lutte contre la Résistance aux Antimicrobiens (RAM) 2019–2023	French	Ministère de la Santé et de l'Hygiène Publique (MSHP) Ministère de l'Élevage et de la Pêche (MEP) Ministère de l'Agriculture (MA) Ministère de l'Environnement, de l'Assainissement et du Développement Durable (MEADD)	Human, animal and environmental health under the One Health approach	https://www.who.int/publications/m/item/mali‐national‐action‐plan‐on‐antimicrobial‐resistance‐2019‐2023
Nigeria	National Action Plan for Antimicrobial Resistance (2017–2022)	English	Federal Ministry of Health (FMOH) Federal Ministry of Agriculture and Rural Development (FMARD) Federal Ministry of Environment (FMEnv) Nigeria Centre for Disease Control (NCDC) (Technical implementation and coordination body)	Implementation of One Health approach focusing on AMR containment in human, animal and environmental health sectors with commitment to AMR surveillance, public awareness and AMR research	https://www.who.int/publications/m/item/nigeria‐national‐action‐plan‐for‐antimicrobial‐resistance
Nigeria	One Health Antimicrobial Resistance National Action Plan 2.0 (2024–2028)	English	Federal Ministry of Health and Social Welfare Federal Ministry of Agriculture and Food Security Federal Ministry of Environment Nigeria Centre for Disease Control (NCDC) (Technical implementation and coordination body)	Addressing AMR across human health, animal health, agriculture and environment through a One Health approach	https://www.who.int/publications/m/item/nigeria‐‐second‐one‐health‐antimicrobial‐resistance‐national‐action‐plan‐2024‐2028
Senegal	Plan d'Action National Multisectoriel de Surveillance et de Lutte Contre les Résistances aux Antimicrobiens (RAM) 2018–2022	French	Ministère de la Santé et de l'Action Sociale (MSAS) Ministère de l'Agriculture Ministère de l'Environnement	Multisectoral One Health approach to combat AMR in human health, animal health and food safety sectors	https://www.who.int/publications/m/item/senegal‐multisectoral‐national‐action‐plan‐against‐antimicrobial‐resistance‐2017
Sierra Leone	National Strategic Plan for Combating Antimicrobial Resistance (2018–2022)	English	Ministry of Health and Sanitation (MOHS) Ministry of Agriculture, Forestry and Food Security (MAFFS) Environment Protection Agency (EPA)	Implementation of One Health approach addressing AMR in human health, agriculture and environmental sectors	https://www.who.int/publications/m/item/sierra‐leone‐national‐strategic‐plan‐for‐combating‐antimicrobial‐resistance
Togo	Plan d'Action National de Lutte Contre la Résistance aux Antimicrobiens (2019–2023)	French	Ministère de la Santé, de l'Hygiène Publique et de l'Accès Universel aux Soins Ministère de l'Agriculture, de l'Élevage et du Développement Rural Ministère de l'Environnement et des Ressources Forestières	One Health approach with focus on surveillance, education, regulation of antibiotic use and capacity strengthening in human, animal and environmental health	https://www.who.int/publications/m/item/Togo‐national‐action‐plan‐against‐antimicrobial‐resistance‐2019‐2023
Benin	Plan d'Action National multisectoriel de lutte contre la Résistance aux Antimicrobiens (2019–2024)	French	Ministère de la Santé Ministère de l'Agriculture, de l'Élevage et de la Pêche Ministère du Cadre de Vie et du Développement Durable	Multisectoral One Health approach addressing human, animal and environmental health through surveillance, education, prevention and rational antimicrobial use	https://www.who.int/publications/m/item/benin‐national‐multisectoral‐action‐plan‐against‐antimicrobial‐resistance‐2019‐2024‐(french)

### 3.3 Inclusion of Vulnerable and Marginalised Populations in AMR NAPs

Table 3 summarises the extent to which the 14 West African NAPs on AMR addressed the needs of vulnerable and marginalised populations. While most NAPs reference general rural communities, healthcare workers, or those with poor healthcare access, explicit identification of highly marginalised groups, such as women, children, displaced persons, or the urban poor, was limited. Only Nigeria's second action plan (2024–2028) directly mentioned specific vulnerable groups and proposed targeted interventions to reduce inequities. Across countries, common interventions included hygiene promotion, infection prevention and control (IPC) and awareness campaigns aimed at rural or general populations. Stakeholder engagement was often multisectoral, involving ministries of health, agriculture, environment and civil society organisations. However, none of the NAPs explicitly integrated equity considerations into AMR governance, monitoring, or evaluation frameworks. Overall, while some plans incorporate inclusive language and community‐level interventions, they fall short of operationalising equity in a systematic or measurable way.

**TABLE 3:** Summary synthesis of inclusion of vulnerable and marginalised populations in National Action Plan in the 14 West African countries.

Country	Vulnerable/marginalised groups mentioned	Specific interventions for these groups	Stakeholder engagement	Equity in AMR governance and monitoring	Overall assessment
Burkina Faso	General vulnerable populations at community level Healthcare workers and agricultural stakeholders	Hygiene awareness campaigns (handwashing, infection prevention) Public communication strategies (radio, posters, campaigns) Training of health workers	Involvement of National AMR Commission, Ministries of Health, Agriculture and Environment Support from community stakeholders and experts	Not explicit	Vulnerable populations are addressed through general interventions, but specific targeted actions for marginalised groups are not clearly detailed
Cabo Verde	Healthcare workers, rural communities and low‐income populations	Continuous training for healthcare workers on infection prevention and control Awareness programmes targeting rural communities and hygiene promotion Support for rational use of antimicrobials in human, animal, and environmental health	Involvement of government ministries, NGOs, community associations and international partners in education and intervention programmes	Not explicit	Robust stakeholder involvement, but limited specific focus on highly vulnerable populations
Côte d'Ivoire	Rural communities, healthcare workers, prescribers	Campaigns for raising awareness on hygiene and infection prevention Continuous training for prescribers and healthcare professionals Surveillance and vaccination programmes targeting infectious diseases	Strong stakeholder involvement of government ministries, NGOs and international agencies in coordination and awareness efforts	Not explicit	Robust focus on healthcare systems, but lack of explicit inclusion of highly vulnerable or marginalised populations
Ghana	Rural communities, healthcare workers, livestock farmers, children, low‐income populations	Public education campaigns on responsible use of antimicrobials targeting general and rural populations Infection prevention and control (IPC) programmes for healthcare workers Safe antibiotic practices and training for livestock farmers to minimise AMR spread Improved access to potable water, sanitation and hygiene in health facilities and rural communities Emphasis on vaccination programmes for disease prevention in children	Multi‐sectoral collaboration involving Ministry of Health, Ministry of Food and Agriculture, Ministry of Environment, Science, Technology and Innovation, Ministry of Fisheries and Aquaculture Development; Civil Society Organisations (CSOs); media partners; WHO; FAO; and the private sector	Not explicit	Ghana's NAP takes a One Health approach with interventions aimed at the general population and sectors (human, animal, environment). However, specific, targeted actions for vulnerable or marginalised populations are not explicitly mentioned
Guinea	Rural communities, healthcare workers, livestock farmers, informal vendors, low‐income populations	Public education campaigns to promote the responsible use of antimicrobials Infection prevention and control (IPC) programmes for healthcare workers Training for livestock farmers and informal vendors on safe antibiotic practices Awareness creation and hygiene promotion in rural areas	Multi‐sectoral engagement involving Ministère de la Santé, Ministère de l'Agriculture, Ministère de l'Environnement and Ministère de l'Élevage, along with support from international partners like WHO, FAO and WOAH	Not explicit	Guinea's NAP follows a One Health approach with broad strategies. However, vulnerable and marginalised groups lack tailored interventions
Niger	Healthcare workers (including veterinarians and paraprofessionals) and rural communities	Training modules on AMR stewardship for health professionals and animal‐health workers Implementation of hygiene promotion campaigns targeting rural areas	National AMR Commission (C‐RAM) and regional branches Ministry focal points in Health, Livestock, Agriculture, Environment Technical & financial partners (WHO, FAO, USAID, AFD, Enabel)	Not explicit	Strong multisectoral governance and broad training/outreach but lacks targeted strategies or monitoring for specific vulnerable or marginalised groups
Guinea‐Bissau	Healthcare workers, rural communities, farmers (livestock and agriculture)	Public education campaigns on the rational use of antimicrobials targeting the general population Training programmes for healthcare workers on Infection Prevention and Control (IPC) and rational prescribing of antibiotics Improved access to water, sanitation and hygiene (WASH) in rural communities to reduce infections Education for farmers and veterinarians on biosafety measures and rational antibiotic use in livestock	Ministério da Saúde Pública (MINSAP) (Lead) Collaboration with Ministério da Agricultura, Ministério do Ambiente and Ministério das Pescas Support from WHO, FAO, WOAH, UNEP, Civil Society and other international partners	Not explicit	The plan follows a strong One Health approach, targeting human health, animal health and the environment. While rural and professional groups are addressed, there is no explicit mention of most marginalised and vulnerable populations
Liberia	Communities with poor access to healthcare Healthcare workers Rural populations People affected by counterfeit drugs	Conducting community‐level awareness campaigns on sanitation, hygiene, and IPC (Infection Prevention and Control) Establishing community waste management protocols and water safety plans Training Community Health Assistants (CHAs) to promote hygiene practices Ensuring equitable access to safe drinking water in rural areas	Multisectoral engagement under the One Health Coordination Platform, involving ministries, technical partners and community‐level stakeholders Engagement of CHAs, traditional leaders and civil society organisations (CSOs) in rural and underserved communities	Not explicit	Strong inclusion of community‐level interventions but limited focus on specific vulnerable populations. Emphasis is on rural areas and healthcare access challenges
Mali	Rural populations Communities with poor access to healthcare or chronic conditions Groups affected by illicit drug sales and counterfeit antibiotics Healthcare workers	Implementation of hygiene promotion campaigns targeting rural areas Development of community education programmes on antimicrobial use and resistance Strengthening healthcare infrastructure to ensure access to potable water and infection control measures	Led by the Groupe de Coordination Multisectorielle Nationale (GCMN) Engagement of ministries (health, agriculture, environment and livestock), technical partners and community‐based organisations	Not explicit	Effective integration of community‐level strategies but limited specificity on detailed vulnerable or marginalised group needs
Nigeria (First Action Plan; National Action Plan for Antimicrobial Resistance [2017–2022])	Rural communities Communities with poor healthcare access Healthcare workers Populations vulnerable to counterfeit medicines	Improving access to healthcare services through community‐based programmes Promoting awareness campaigns targeting antibiotic misuse and hygiene in rural communities Strengthening regulations to combat counterfeit medicines and ensure availability of quality antimicrobials	Engagement of government ministries (health, agriculture, environment) Collaboration with CSOs, NGOs, traditional/religious leaders, and private sector Supported by partners such as WHO and FAO	Not explicit	The plan addresses rural and underserved populations but lacks tailored strategies for many other marginalised or vulnerable groups
Nigeria (Second Action Plan; One Health Antimicrobial Resistance National Action Plan 2.0 [2024–2028])	Women Children Rural and displaced populations Communities with limited access to healthcare and clean water Healthcare workers	Promoting equitable access to preventive measures, diagnosis, and treatment Focused interventions addressing gender inequalities in AMR Enhancing access to vaccination, IPC and WASH programmes in underserved areas	Collaboration with government sectors and international partners like WHO, FAO, WOAH, UNEP Engagement of CSOs, community leaders, and private sector for grassroots inclusion	Not explicit	The plan demonstrates a strong commitment to addressing AMR inequities, especially in vulnerable and underserved populations. Monitoring and funding will determine its effectiveness
Senegal	Rural populations Communities with poor healthcare access Healthcare workers Food‐insecure groups	Improving access to antibiotic surveillance and infection prevention in rural health facilities Awareness programmes for rational antibiotic use targeting healthcare workers, rural, and food‐insecure communities	Collaboration among government ministries (Health, Agriculture, Environment) Support from WHO and FAO Engagement of community leaders and CSOs	Not explicit	The plan addresses AMR broadly but lacks detailed, tailored interventions for vulnerable and marginalised groups
Sierra Leone	Rural communities Communities with poor access to healthcare Healthcare workers Groups exposed to environmental contamination	Strengthening infection prevention and control (IPC) at community and health facility levels Promoting awareness campaigns on hygiene and proper antimicrobial use Developing waste management systems to prevent environmental AMR contamination	Collaboration between MOHS, MAFFS and EPA Partnerships with WHO, FAO and local stakeholders, including CSOs	Not explicit	The plan includes community‐level strategies but lacks targeted interventions for vulnerable and marginalised groups
Togo	Rural communities Healthcare workers Communities with limited access to healthcare Groups affected by counterfeit medicines	Community‐based awareness campaigns targeting rational antibiotic use Improving access to healthcare infrastructure and hygiene facilities Strengthening regulations to combat counterfeit antibiotics	Collaboration between health, agriculture and environment ministries Involvement of community leaders, CSOs and technical partners like WHO and FAO	Not explicit	The plan highlights rural communities but lacks tailored strategies for most vulnerable and marginalised groups
Benin	Rural communities Healthcare workers Communities with limited healthcare access	Promoting awareness campaigns targeting antimicrobial misuse and hygiene practices in underserved areas Strengthening healthcare infrastructure and improving access to IPC (infection prevention and control) measures	Collaboration between health, agriculture and environment ministries Engagement with community leaders, CSOs, WHO, FAO and local institutions	Not explicit	The plan addresses rural and underserved populations but lacks tailored interventions for vulnerable and marginalised groups

### 3.4 Inclusion Mapping of Vulnerable and Marginalised Groups

**FIGURE 2:**
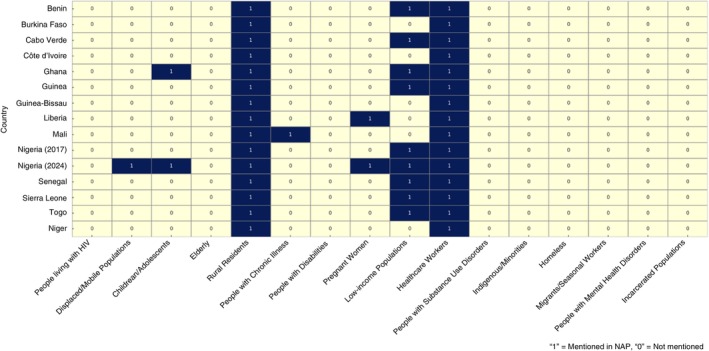
Mapping the inclusion of vulnerable and marginalised populations in AMR National Action Plans across 14 West African countries.

Figure 2 presents a comparative overview of the extent to which Antimicrobial Resistance NAPs from 14 West African countries explicitly acknowledge vulnerable and marginalised populations. The analysis shows that healthcare workers and rural residents were the only groups consistently mentioned across all countries. By contrast, people with chronic illnesses were only referenced in one country (Mali) and (pregnant) women appeared in just two NAPs (Liberia and Nigeria 2024). Notably, no country's NAP made any reference to people living with HIV, people with disabilities, the elderly, or populations experiencing mental health disorders, homelessness, incarceration, or seasonal migration. Only Nigeria's second NAP (2024–2028) demonstrated a more inclusive approach, referencing six vulnerable groups, including children/adolescents, displaced populations, pregnant women, healthcare workers, rural residents and low‐income populations. This visual disparity reveals that most West African NAPs do not systematically integrate an equity lens, missing opportunities to address the specific AMR risks faced by structurally disadvantaged populations.

## 4 Discussion

### 4.1 Unequal Burdens of AMR

AMR is widely recognised as one of the most pressing global health threats of our time, but its impacts are not borne equally. Although AMR can affect any population, the burden falls disproportionately on socially and economically marginalised groups—such as people living in rural and remote communities, those residing in informal urban settlements, incarcerated individuals, displaced populations and those experiencing poverty or chronic illness. These populations often experience a convergence of structural disadvantages: limited access to healthcare services, inadequate water and sanitation infrastructure, poor living conditions and reliance on informal markets for medicine, including substandard or falsified antimicrobials [19, 20, 21].

Our systematic analysis of AMR NAPs from 14 West African countries highlights a critical shortfall in how equity considerations are integrated into national AMR policies. While all countries adopt a multisectoral “One Health” framework on paper, addressing AMR across human, animal and environmental domains, few plans offer clear, actionable pathways to address the distinct vulnerabilities and needs of high‐risk populations. This omission has significant implications for the effectiveness and fairness of national responses.

### 4.2 Gaps in Global and Regional Guidance

The global policy foundation for national AMR responses was laid by the 2015 World Health Organization GAP on AMR, which urged countries to adopt national strategies aligned with five overarching objectives [10]. While this framework was instrumental in galvanising political commitment and shaping the architecture of national plans, it paid limited attention to the social determinants of AMR [11]. Concepts such as poverty, gender inequity, disability, migration, and incarceration were not mentioned in the original GAP, leaving a critical gap in how countries conceptualised vulnerability in their NAPs. Subsequent technical guidance from WHO, such as the guidance on gender inequalities and AMR [22], the people‐centred approach to addressing AMR in human health [23] and the WHO Implementation Handbook for National Action Plans on Antimicrobial Resistance: Guidance for the Human Health Sector [24], has sought to correct this oversight by explicitly encouraging equity‐sensitive approaches. However, the uptake of these recommendations in national policy documents has been uneven. Our findings demonstrate that while many NAPs contain generic language on public awareness, hygiene promotion and healthcare worker training, very few articulate structured strategies to identify and respond to population‐specific inequities. Only Nigeria's second NAP (2024–2028) explicitly references women, children and displaced communities, illustrating a broader regional trend of symbolic rather than substantive equity inclusion.

### 4.3 Epistemic Injustice and Excluded Voices

A growing body of literature suggests that the limited integration of equity into national AMR policies is not merely an oversight but reflects a deeper form of epistemic injustice. This refers to the structural exclusion of certain voices, knowledge systems and lived experiences from research and policy processes. As Cheah and colleagues argue, global AMR research and governance continue to be shaped largely by actors from high‐income countries and biomedical disciplines, while the perspectives and priorities of communities most affected by AMR, particularly in LMICs, are often marginalised or ignored [25]. This has important consequences for vulnerable groups such as displaced persons, rural residents, informal workers and people living with disabilities or chronic illnesses. These populations are frequently excluded from formal surveillance systems, their lived realities are under‐represented in global evidence and the types of knowledge they possess, including oral traditions and non‐English‐language materials, are often undervalued in decision‐making. When such groups are not involved in setting research agendas or designing interventions, national AMR strategies are less likely to address the specific conditions that heighten their risk, such as inadequate sanitation, lack of affordable diagnostics, or reliance on informal medicine vendors. Addressing epistemic injustice requires intentional inclusion of diverse perspectives, stronger representation of researchers from low‐resource settings and recognition of community‐based and experiential knowledge as valid forms of evidence in AMR policymaking. These strategic directions are outlined in greater detail in Table S2, which presents practical recommendations across governance, surveillance, health communication, service delivery and capacity building.

### 4.4 Vulnerability Is Not Monolithic

It is also essential to recognise that vulnerability to AMR is not monolithic; different population groups face distinct risks and barriers that require tailored responses [25]. Children, for example, may not have access to age‐appropriate antibiotic formulations and paediatric AMR trends are often poorly documented in national surveillance systems. People living with HIV may experience recurrent infections requiring prophylactic antibiotics, which heightens the risk of resistance if care is fragmented or substandard. Displaced populations and seasonal workers may lack documentation or language fluency, making it difficult to navigate healthcare systems or understand treatment protocols. Pregnant women face unique immunological and perinatal risks, while incarcerated individuals often live in overcrowded environments with weak infection control. Yet, our analysis found that these nuances are largely absent from West African NAPs. Addressing these omissions requires broad shifts in how AMR policy is designed and implemented, including the delivery of tailored services for at‐risk groups, more inclusive surveillance systems that capture disaggregated data and greater participation of vulnerable populations in policy processes [26]. A more detailed set of recommendations for 16 distinct vulnerable groups, ranging from rural communities and healthcare workers to people with disabilities, substance use disorders, or mental health conditions, is provided in Table S3.

### 4.5 Intersectionality and Overlapping Disadvantages

An equity‐oriented approach to AMR must also recognise that some individuals face multiple, overlapping disadvantages that increase their risk of exposure to resistant infections and limit their ability to access effective treatment. For example, a woman living with a disability in an informal settlement may encounter barriers not only related to her physical condition but also to poverty, gender‐based discrimination and lack of secure housing. These layered forms of exclusion often remain invisible when policies focus on population groups in isolation. Understanding how characteristics such as age, gender, income level, disability, housing status and migration history interact is essential to designing responsive and inclusive AMR strategies. Intersectional thinking allows policymakers to identify those who are most at risk of being overlooked in surveillance, prevention and treatment efforts [27, 28, 29, 30]. By acknowledging these overlapping vulnerabilities, NAPs can better align with the realities of those who face the highest burden of AMR and the lowest access to care.

### 4.6 A Call to Action for Inclusive AMR Governance

In light of these findings, there is an urgent need for a paradigm shift in how equity is conceptualised and operationalised within AMR strategies. Countries must explicitly name vulnerable and marginalised populations in their NAPs, develop dedicated indicators to track their inclusion and allocate financial and institutional resources to address their needs. Equally important is the need to foster participatory governance structures that include civil society, patient advocacy groups and community‐based organisations in both the design and evaluation of national plans. Equity must also be embedded in data systems: routine disaggregation by sex, age, geographic location and socioeconomic status is essential for identifying gaps and informing course correction. As countries in West Africa and beyond prepare to update their AMR strategies in the coming years, there is a unique opportunity to institutionalise equity as a core principle of health security.

Moving from principle to practice requires coordinated action at multiple levels. At the national level, health authorities should identify and name priority populations in their NAPs, while ensuring that public health messaging, service delivery and access strategies are inclusive of underserved groups. They should also disaggregate surveillance and prescribing data to ensure that policies are responsive to those most at risk of exclusion. At the regional level, ECOWAS and WAHO can play a crucial role in developing equity guidelines, supporting harmonisation of disaggregated AMR data and creating platforms for sharing good practices. Africa CDC can strengthen this ecosystem further by integrating equity into One Health capacity building, incorporating marginalised groups into regional AMR risk assessments and ensuring equity is embedded in the next generation of NAPs. Civil society and community‐based organisations should be empowered to advocate for vulnerable groups, monitor access to quality antimicrobials and information and provide community‐level feedback on implementation. International partners, including WHO, FAO, UNEP and WOAH, can support countries by aligning technical assistance with local equity needs, funding context‐relevant research and helping to develop equity‐sensitive monitoring frameworks. Finally, researchers and academia have a critical role in generating policy‐relevant evidence, evaluating how interventions affect different social groups and building local capacity for mixed‐methods policy analysis. The full set of detailed actions for each stakeholder group is provided in Table S4, which offers a comprehensive call to action for embedding equity in AMR governance across West Africa.

### 4.7 Strengths and Limitations

This study presents the first systematic equity‐focused analysis of Antimicrobial Resistance National Action Plans across 14 countries in the ECOWAS region, offering a unique and timely contribution to both regional and global health policy discourse. A key strength of this research lies in its use of a structured, theory‐informed analytic framework, which was grounded in established models of health equity and refined through expert input. This allowed for a consistent and comprehensive assessment of how NAPs recognise and respond to the needs of vulnerable and marginalised populations. The inclusion of a diverse range of population groups, ranging from rural communities and people living with HIV to those with disabilities or in incarceration, ensured that the equity lens applied was intersectional and reflective of real‐world disparities. Moreover, the multilingual review approach, involving coders fluent in English, French and Portuguese, helped preserve the nuance of non‐English policy documents and reduce the risk of misinterpretation. An additional strength was the reflexive, consensus‐driven coding process, which enhanced analytical rigour and minimised individual bias.

However, the study also has several limitations that must be acknowledged. First, the analysis is based solely on publicly available policy documents and does not include primary data on implementation practices or outcomes. As a result, there may be discrepancies between what is stated in the NAPs and what is operationalised in practice. Second, while the use of standardised extraction templates improved consistency, the subjective interpretation of policy language, especially in vague or aspirational statements, may still have introduced coder bias, despite our efforts to mitigate this through cross‐checking and team debriefings. Third, the absence of publicly available NAPs from one ECOWAS member state (The Gambia) limits the regional representativeness of the sample, although the 14 countries included still provide a robust overview of policy trends. Despite these limitations, the study offers useful insights into existing policy gaps and practical opportunities for embedding equity in AMR response across West Africa.

## 5 Conclusion

AMR is a social and political challenge that demands equity‐centred responses. This study has demonstrated that while all 14 reviewed ECOWAS countries have developed NAPs aligned with the One Health approach, the vast majority fall short of systematically identifying or addressing the needs of vulnerable and marginalised populations. Equity is often referenced in general terms but remains poorly integrated into governance structures, data systems, intervention design and monitoring frameworks. This lack of operational focus risks exacerbating health disparities, weakening AMR containment efforts and undermining public trust in national and regional responses. Our findings point to a critical opportunity for policy transformation. As West African countries prepare to update or expand their AMR strategies, there is both an ethical and practical imperative to embed equity at every stage of the policy cycle. This includes explicitly naming at‐risk populations, developing targeted interventions, strengthening participatory governance and tracking disaggregated data to ensure accountability. Regional bodies such as ECOWAS and Africa CDC have an important role to play in harmonising equity standards, supporting capacity‐building and mobilising political commitment across member states. National health authorities, in turn, must ensure that AMR plans reflect the lived realities of those most affected by resistance—whether due to geography, gender, income, displacement or structural exclusion.

An equity‐informed approach is essential to achieving the overarching goals of the GAP on AMR and the SDGs, particularly the commitment to universal health coverage and leaving no one behind. The path forward requires deliberate attention to who is being served, who is being overlooked and how health systems can be restructured to protect the most vulnerable. In confronting AMR, equity must not be seen as an optional add‐on, but as a central pillar of resilient, ethical and sustainable public health policy.

## Conflicts of Interest

The authors declare no conflicts of interest.

## Supporting information


**Data S1:** tmi70037‐sup‐0001‐supinfo.docx.

## Data Availability

The data supporting the findings of this study consist of publicly available Antimicrobial Resistance National Action Plans. All documents analysed are cited within the manuscript and accessible at: https://www.who.int/teams/surveillance‐prevention‐control‐amr/national‐action‐plan‐monitoring‐evaluation/library‐of‐national‐action‐plans and through related country‐specific pages. No new data were generated during this study.
