# Supplementary material for: An Equity‐Focused Systematic Analysis of Antimicrobial Resistance National Action Plans in 14 West African Countries
Source: Trop Med Int Health. 2025 Sep 20;30(12):1295–312. doi: 10.1111/tmi.70037 (PMC12675314; doi:10.1111/tmi.70037)
Supplement: Supplementary file 1 — Data S1: tmi70037‐sup‐0001‐supinfo.docx. [file TMI-30-1295-s001.docx]

***Supplementary File***

**An Equity-Focused Systematic Analysis of Antimicrobial Resistance National Action Plans in 14 West African Countries**

Yusuff Adebayo Adebisi¹˒²,*** Isaac Olushola Ogunkola¹˒³, Adeola Bamisaiye⁴, Aminat Olaitan Adebayo⁵, Noah Sesay⁶, Kwasi Yelarge⁷, Don Eliseo Lucero-Prisno III¹˒⁸

¹ Global Health Focus, Abuja, Nigeria; y.adebisi.1@research.gla.ac.uk
² College of Social Sciences, University of Glasgow, Glasgow, UK
³ Nuffield Department of Population Health, University of Oxford, Oxford, UK
⁴ Centre for Tropical Medicine and Global Health, University of Oxford, Oxford, UK
⁵ Department of Agricultural Extension and Rural Development, Faculty of Agriculture, University of Ibadan, Ibadan, Nigeria
⁶ Ministry of Health of Sierra Leone, Ola During Children’s Hospital, Freetown, Sierra Leone
⁷ Faculty of Pharmacy and Pharmaceutical Sciences, Kwame Nkrumah University of Science and Technology, Ghana
⁸ Department of Global Health and Development, London School of Hygiene and Tropical Medicine, London, United Kingdom

*Correspondence: [y.adebisi.1@research.gla.ac.uk](mailto:y.adebisi.1@research.gla.ac.uk)

**Supplementary Table 1: AMR Burden and Why Equity Matters in 14 West African Countries**

| **Country** | **AMR Burden in 2019 [5]** | **Why Equity Matters** |
| --- | --- | --- |
| Burkina Faso | -Burkina Faso recorded 7,000 deaths attributable to AMR and 30,500 deaths associated with AMR, placing it among the top 10 countries globally for age-standardized mortality rates.  -Within Western Sub-Saharan Africa, Burkina Faso ranks 2nd highest in age-standardized AMR mortality, surpassing deaths from other major diseases such as neglected tropical diseases, malaria, cardiovascular, maternal/neonatal disorders, and enteric infections.  - Five main pathogens drive the high AMR burden—*Klebsiella pneumoniae* (6,200 deaths), *Streptococcus pneumoniae* (5,800), *Staphylococcus aureus* (4,800), *Escherichia coli* (4,300), and *Pseudomonas aeruginosa* (1,500)—primarily causing lower respiratory and thoracic infections, as well as bloodstream infections. | High burden in rural and underserved areas highlights need for equitable access to AMR diagnostics and care. |
| Carbo Verde | -Cabo Verde recorded 79 deaths attributable to AMR and 321 deaths associated with AMR, ranking 85th globally for age-standardized AMR mortality but lowest in Western Sub-Saharan Africa.  -AMR-related deaths in Cabo Verde exceed those from diabetes, kidney diseases, digestive diseases, self-harm, neurological disorders, and unintentional injuries.  -Five key pathogens drive the AMR burden: *Acinetobacter baumannii* (47 deaths), *Streptococcus pneumoniae* (46), *Staphylococcus aureus* (44), *Klebsiella pneumoniae* (43), and *Escherichia coli* (43), causing primarily bloodstream, respiratory, and intraabdominal infections. | Marginalized island and low-income populations may still face access barriers despite low overall burden. |
| Cote d’Ivoire | -Côte d’Ivoire recorded 5,800 AMR-attributable deaths and 24,800 AMR-associated deaths, ranking 27th globally but 9th lowest in Western Sub-Saharan Africa for age-standardized AMR mortality.  -AMR deaths in Côte d’Ivoire exceed those from respiratory infections, tuberculosis, cardiovascular diseases, and HIV/AIDS.  -Five key pathogens contribute to the burden: *Klebsiella pneumoniae* (4,700), *Streptococcus pneumoniae* (4,400), *Escherichia coli* (3,400), *Staphylococcus aureus* (3,300), and *Group B Streptococcus* (1,500), primarily causing respiratory, bloodstream, intraabdominal, and sexually transmitted infections. | Rural and peri-urban communities may be disproportionately affected due to limited health infrastructure. |
| Ghana | -Ghana recorded 5,900 AMR-attributable deaths and 25,300 AMR-associated deaths, ranking 36th globally and 6th lowest in Western Sub-Saharan Africa for age-standardized AMR mortality.  -AMR deaths in Ghana exceed those from neglected tropical diseases, malaria, neoplasms, maternal/neonatal disorders, HIV/AIDS, and diabetes/kidney diseases.  -Five key pathogens drive the burden: *Klebsiella pneumoniae* (4,600), *Streptococcus pneumoniae* (4,200), *Staphylococcus aureus* (3,700), *Escherichia coli* (3,600), and *Pseudomonas aeruginosa* (1,600), primarily causing respiratory, bloodstream, and intraabdominal infections. | Socioeconomic inequalities in healthcare access could worsen outcomes among low-income or rural populations. |
| Guinea | -Guinea recorded 4,000 AMR-attributable deaths and 17,400 AMR-associated deaths, ranking among the top 10 globally and 3rd highest in Western Sub-Saharan Africa for age-standardized AMR mortality.  -AMR deaths in Guinea exceed those from cardiovascular diseases, maternal/neonatal disorders, neglected tropical diseases, malaria, enteric infections, and neoplasms.  -Five key pathogens contribute to the burden: *Streptococcus pneumoniae* (4,700), *Klebsiella pneumoniae* (2,700), *Escherichia coli* (2,000), *Staphylococcus aureus* (1,400), and *Group B Streptococcus* (1,300), primarily causing respiratory, central nervous system, bloodstream, and cardiac infections. | Conflict-affected and remote areas face additional challenges in access to timely diagnosis and effective treatment. |
| Niger | - Niger experienced 7,100 deaths attributable to AMR and 31,300 deaths associated with AMR healthdata.org  - Ranked 16th highest globally (out of 204 countries) for age-standardized AMR mortality rate, and 6th highest within Western Sub-Saharan Africa  - Driven by five main pathogens, *Streptococcus pneumoniae* (7,800 associated deaths), *Klebsiella pneumoniae* (6,100), *Escherichia coli* (4,500), *Staphylococcus aureus* (3,300), and Group B Streptococcus (2,200), primarily causing lower respiratory, thoracic, CNS, bloodstream and cardiac infections. | Rural and underserved regions with limited robust health infrastructure amplify inequitable health outcomes. |
| Guinea-Bissau | -Guinea-Bissau recorded 413 AMR-attributable deaths and 1,800 AMR-associated deaths, ranking among the top 10 globally and 1st highest in Western Sub-Saharan Africa for age-standardized AMR mortality.  -AMR deaths in Guinea-Bissau exceed those from maternal/neonatal disorders, enteric infections, HIV/AIDS, other infectious diseases, and neoplasms.  -Five key pathogens drive the burden: *Klebsiella pneumoniae* (350), *Streptococcus pneumoniae* (325), *Staphylococcus aureus* (272), *Escherichia coli* (260), and *Acinetobacter baumannii* (102), primarily causing respiratory, bloodstream, and intraabdominal infections. | Fragile health systems and exclusion of poor, rural communities amplify inequitable health outcomes. |
| Liberia | -Liberia recorded 772 AMR-attributable deaths and 3,400 AMR-associated deaths, ranking 41st globally and 4th lowest in Western Sub-Saharan Africa for age-standardized AMR mortality.  -AMR deaths in Liberia exceed those from respiratory infections, tuberculosis, maternal/neonatal disorders, enteric infections, HIV/AIDS, and neoplasms.  -Five key pathogens drive the burden: *Klebsiella pneumoniae* (669), *Escherichia coli* (533), *Streptococcus pneumoniae* (503), *Staphylococcus aureus* (464), and *Pseudomonas aeruginosa* (215), primarily causing respiratory, bloodstream, and intraabdominal infections. | Recent health system disruptions make equitable inclusion of rural and underserved populations essential. |
| Mali | -Mali recorded 7,100 AMR-attributable deaths and 29,700 AMR-associated deaths, ranking 26th globally and 10th highest in Western Sub-Saharan Africa for age-standardized AMR mortality.  -AMR deaths in Mali exceed those from respiratory infections, tuberculosis, enteric infections, cardiovascular diseases, neglected tropical diseases, malaria, and nutritional deficiencies.  -Five key pathogens drive the burden: *Klebsiella pneumoniae* (6,500), *Escherichia coli* (4,400), *Streptococcus pneumoniae* (4,200), *Staphylococcus aureus* (3,500), and *Group B Streptococcus* (1,900), primarily causing respiratory, bloodstream, and central nervous system infections. | AMR burden exacerbated in conflict zones and regions with poor healthcare access and chronic insecurity. |
| Nigeria | -Nigeria recorded 64,500 AMR-attributable deaths and 263,400 AMR-associated deaths, ranking 20th globally and 7th highest in Western Sub-Saharan Africa for age-standardized AMR mortality.  -AMR deaths in Nigeria exceed those from enteric infections, respiratory infections, tuberculosis, maternal/neonatal disorders, neglected tropical diseases, malaria, and cardiovascular diseases.  -Five key pathogens drive the burden: *Streptococcus pneumoniae* (54,300), *Klebsiella pneumoniae* (44,300), *Escherichia coli* (38,200), *Staphylococcus aureus* (32,300), and G*roup B Streptococcus* (16,600), primarily causing respiratory, central nervous system, bloodstream, and intraabdominal infections. | Massive inequality in healthcare distribution; AMR burden could widen urban-rural and wealth-health gaps. |
| Senegal | -Senegal recorded 2,700 AMR-attributable deaths and 11,900 AMR-associated deaths, ranking 43rd globally and 3rd lowest in Western Sub-Saharan Africa for age-standardized AMR mortality.  -AMR deaths in Senegal exceed those from maternal/neonatal disorders, respiratory infections, tuberculosis, neoplasms, enteric infections, and neglected tropical diseases/malaria.  -Five key pathogens drive the burden: *Klebsiella pneumoniae* (2,300), *Staphylococcus aureus* (1,900), *Escherichia coli* (1,900), *Streptococcus pneumoniae* (1,700), and *Acinetobacter baumannii* (779), primarily causing respiratory, bloodstream, and intraabdominal infections. | Coastal and rural populations require equitable attention to ensure AMR interventions reach all segments. |
| Sierra-Leone | -Sierra Leone recorded 2,200 AMR-attributable deaths and 9,700 AMR-associated deaths, ranking 15th globally for age-standardized AMR mortality and 5th highest in Western Sub-Saharan Africa for age-standardized AMR mortality.  -AMR deaths in Sierra Leone exceed those from cardiovascular diseases, maternal/neonatal disorders, enteric infections, neoplasms, and other non-communicable diseases.  -Five key pathogens contribute to the burden: *Streptococcus pneumoniae* (2,000), *Klebsiella pneumoniae* (1,900), *Staphylococcus aureus* (1,300), *Escherichia coli* (1,300), and *Pseudomonas aeruginosa* (558), primarily causing respiratory, bloodstream, cardiac, and central nervous system infections. | Vulnerable post-conflict systems necessitate inclusive AMR planning for internally displaced and poor communities. |
| Togo | -Togo recorded 1,500 AMR-attributable deaths and 6,300 AMR-associated deaths, ranking 32nd globally and 7th lowest in Western Sub-Saharan Africa for age-standardized AMR mortality.  -AMR deaths in Togo exceed those from neglected tropical diseases/malaria, maternal/neonatal disorders, neoplasms, HIV/AIDS, and digestive diseases.  -Five key pathogens drive the burden: *Klebsiella pneumoniae* (1,200), *Staphylococcus aureus* (1,000), *Streptococcus pneumoniae* (974), *Escherichia coli* (963), and *Pseudomonas aeruginosa* (353), primarily causing respiratory, bloodstream, and intraabdominal infections. | Urban poor and rural dwellers may be disproportionately affected without targeted AMR services and surveillance. |
| Benin | -Benin recorded 3,100 AMR-attributable deaths and 13,900 AMR-associated deaths, ranking 21st globally and 8th highest in Western Sub-Saharan Africa for age-standardized AMR mortality.  -AMR deaths in Benin exceed those from maternal/neonatal disorders, neglected tropical diseases/malaria, respiratory infections, tuberculosis, cardiovascular diseases, and enteric infections.  -Five key pathogens drive the burden: *Klebsiella pneumoniae* (2,700), *Streptococcus pneumoniae* (2,600), *Staphylococcus aureus* (2,000), *Escherichia coli* (1,900), and *Pseudomonas aeruginosa* (772), primarily causing respiratory, bloodstream, and central nervous system infections. | Weak infrastructure in underserved regions may worsen impact among low-income populations without equity-focused action. |

**Supplementary Table 2. Cross-Cutting Recommendations to Advance Equity in National Antimicrobial Resistance Action Plans**

| **Strategic Area** | **Recommendation for Inclusive AMR NAPs** |
| --- | --- |
| Governance and Leadership | Include equity and vulnerability in the vision, objectives, and guiding principles of the AMR National Action Plan. |
| Participation and Representation | Ensure vulnerable populations and civil society groups are represented on national AMR technical working groups and policy review committees. |
| Surveillance Systems | Collect and disaggregate AMR and antimicrobial-use data by age, gender, location (rural/urban), and where possible by socioeconomic or disability status. |
| Health Communication | Translate AMR education materials into local languages; use culturally appropriate communication channels (e.g., radio dramas, community forums). |
| Access to Services | Ensure that essential diagnostics and antimicrobials are available and affordable in rural, low-income, and mobile populations. |
| Monitoring and Evaluation | Track equity indicators (e.g., who is benefiting from AMR interventions) in mid-term and final evaluations of the NAP. |
| Capacity Building | Train healthcare workers and community health volunteers on equity-sensitive AMR practices, including respectful care and inclusive outreach. |

**Supplementary Table 3. Recommendations for Inclusion of Vulnerable and Marginalised Populations in Inclusive AMR National Action Plans**

| **Population Group** | **Recommendations** |
| --- | --- |
| **People living with HIV** | National AMR plans should incorporate data from HIV treatment centres by linking laboratory records of opportunistic infections, such as tuberculosis and pneumonia, to the national surveillance system.  To promote rational prescribing, HIV clinicians should receive tailored training on antimicrobial stewardship principles, including when to switch from first-line to second-line therapies in cases of resistance.  Furthermore, advocacy organisations representing people living with HIV and key populations of HIV should be actively involved in the design of public awareness materials to ensure messaging is inclusive and relevant to this population. |
| **Displaced or Mobile Populations (refugees, internally displaced persons, cross-border traders)** | Mobile health units staffed by community health workers can be deployed to camps and transit hubs to offer point-of-care testing and access to quality-assured antibiotics.  Communication materials, including leaflets and radio announcements, must be translated into the major languages spoken by displaced groups and use clear visuals or pictograms to accommodate low-literacy audiences. |
| **Children and Adolescents** | AMR education should be integrated into existing school health curricula or through extracurricular health clubs, using age-appropriate materials.  The national essential medicines list must ensure the availability of child-friendly formulations, such as syrups or dispersible tablets, along with simplified dosage charts by weight category.  Additionally, paediatric wards in major hospitals should report antimicrobial resistance data separately to allow for the monitoring of trends specific to children and adolescents.  Routine childhood vaccination coverage should be prioritised and monitored, particularly for vaccine-preventable infections that drive antimicrobial use |
| **Older Adults** | Require long-term-care facilities and geriatric wards to implement infection-prevention audits and antimicrobial-stewardship ward rounds at least quarterly.  Train family caregivers and care-home staff on proper antibiotic course completion.  Offer publicly funded pneumococcal and influenza vaccination for citizens above 60 years to reduce infection pressure. |
| **Rural Residents** | Expand district laboratories with basic culture and antimicrobial-susceptibility-testing capability  Establish specimen-transport pilots in high-priority rural districts to connect peripheral clinics to regional laboratories.  Subsidise transport and wholesale prices so rural pharmacies can stock quality-assured antibiotics at the same price as urban outlets.  Air AMR radio programmes on local stations at peak farming hours and integrate messages into existing agricultural-extension meetings. |
| **People with Chronic Illnesses (diabetes, kidney disease, cancer, asthma)** | Incorporate antimicrobial-resistance risk assessments into non-communicable-disease clinic checklists (for example, ask about repeated antibiotic courses or indwelling devices).  Adapt antibiotic-prophylaxis protocols for dialysis and chemotherapy to reflect national resistance patterns.  Encourage selected tertiary hospitals to record antibiotic treatment and infection-related complications among patients with chronic diseases as a pilot surveillance initiative. |
| **People with Disabilities** | Conduct an accessibility audit of health services: ramps, accessible toilets, sign-language interpretation, large-print posters, and clear way-finding signs.  Develop Braille and easy-read leaflets, and record sign-language video messages about rational antibiotic use and infection prevention.  Invite representatives from disability-rights organisations to sit on national AMR steering committees and review public-education campaigns for inclusivity. |
| **Pregnant Women** | Add antibiotic-stewardship counselling and screening for urinary-tract infection to standard antenatal-care check-ups.  Train midwives and obstetricians in evidence-based antibiotic prophylaxis for caesarean section and other obstetric procedures.  Monitor maternal and newborn sepsis cases and publish annual antimicrobial-resistance profiles of the pathogens involved. |
| **Low-Income Populations / Urban Poor** | Expand access to essential health services by including diagnostics and first-line antibiotics in national health insurance schemes or community-based health coverage programmes.  Implement water, sanitation and hygiene upgrades, public hand-washing stations, latrine construction and safe-water kiosks, in informal settlements as part of the AMR plan.  Engage local leaders and pharmacy associations to raise awareness of the risks of informal antibiotic markets and explore community-led monitoring strategies.  Provide non-judgemental counselling on antibiotic adherence and risks of sharing leftover antibiotics. |
| **Healthcare Workers** | Make completion of an online or in-person antimicrobial-stewardship course a requirement for professional licence renewal.  Provide frontline staff with appropriate personal protective equipment, seasonal vaccination, and post-exposure prophylaxis where indicated.  Include strong infection prevention and control as a key performance indicator for health-facility accreditation. |
| **People with Substance-Use Disorders** | Train staff at harm-reduction centres (needle-exchange sites, opioid-substitution-therapy clinics) to recognise soft-tissue infections early and refer for culture and targeted treatment.  Partner with community-based organisations that work with people who use drugs to co-design outreach materials. |
| **People with Mental Health Disorders** | Develop inclusive, non-stigmatising messaging to support antibiotic adherence and combat misconceptions.  Ensure continuity of care by coordinating antibiotic prescribing and follow-up across psychiatric and general health services, to avoid inappropriate antibiotic use and missed doses that contribute to resistance. |
| **Incarcerated Populations** | AMR national plans should explicitly include prisons and detention facilities in their infection prevention strategies. This includes mandating access to basic hygiene infrastructure, first-line antibiotics, and standard infection control protocols within correctional health services.  Health units in prisons should report AMR cases to national surveillance systems to ensure comprehensive data coverage.  Incarcerated individuals should not be excluded from national guidelines or funding allocations for AMR prevention and treatment. |
| **Indigenous or Minority Populations** | Engage traditional and faith leaders in developing culturally appropriate antibiotic-use messages delivered through community gatherings and local media.  Translate patient information sheets into minority languages and use storytellers or drama groups during village health outreaches.  Ensure disaggregated data collection by ethnic group in AMR surveillance to reveal inequities and trigger corrective actions. |
| **Homeless Populations** | Offer drop-in infection-prevention services and wound-care clinics at shelters and day centres.  Equip outreach nurses to provide on-site antibiotic therapy and follow-up to ensure treatment completion.  Integrate targeted AMR education into existing programmes for people living on the street or the slum dwellers |
| **Migrants and Seasonal Workers** | Develop visual, low-literacy materials on safe antibiotic use and hygiene in local languages, distributed through labour recruiters, transport hubs, and religious or community leaders. |

**Supplementary Table 4. Call to Action: Embedding Equity in AMR Governance Across West Africa**

| **Stakeholder** | **Recommended Actions** | **Equity Focus** |
| --- | --- | --- |
| **National Health Authorities** | - Identify and name priority populations in AMR NAPs (e.g. rural residents, displaced people, people with disabilities)  - Ensure public health messaging and access strategies are inclusive of underserved groups  - Disaggregate surveillance and prescribing data by gender, age, location, and social group | Ensure that policies are responsive to the needs of those most at risk of AMR exposure and exclusion |
| **ECOWAS & WAHO** | - Develop regional equity guidelines for AMR planning and implementation  - Coordinate regional platforms for sharing equity-sensitive good practices  - Support harmonisation of disaggregated AMR data across member states | Promote consistency in equity standards and regional accountability mechanisms |
| **Africa CDC** | - Integrate equity into One Health capacity-building initiatives  - Include marginalised groups in regional AMR risk assessments  - Support countries to embed equity in the next generation of NAPs | Strengthen the regional ecosystem for inclusive AMR governance |
| **Civil Society & Community Organisations** | - Advocate for inclusion of vulnerable groups in AMR technical working groups  - Monitor local access to quality antimicrobials and health information  - Provide feedback on NAP implementation through community platforms | Amplify the voices and needs of underserved populations |
| **International Partners (WHO, FAO, UNEP, WOAH)** | - Provide technical assistance for equity-sensitive monitoring and evaluation  - Fund context-relevant research on AMR and social determinants  - Align support with both global norms and local equity needs | Bridge global standards with local realities to reduce inequities |
| **Researchers & Academia** | - Conduct policy-relevant research on equity gaps in AMR service delivery  - Evaluate the impact of AMR interventions on different social groups  - Build local capacity for mixed-methods policy analysis | Generate evidence to guide equity-informed AMR decision-making |
